# Eplerenone Implantation Improved Adipose Dysfunction Averting RAAS Activation and Cell Division

**DOI:** 10.3389/fendo.2020.00223

**Published:** 2020-04-21

**Authors:** Andrea Vecchiola, Cristóbal A. Fuentes, Isidora Solar, Carlos F. Lagos, Maria Cecilia Opazo, Natalia Muñoz-Durango, Claudia A. Riedel, Gareth I. Owen, Alexis M. Kalergis, Carlos E. Fardella

**Affiliations:** ^1^Department of Endocrinology, School of Medicine, Pontificia Universidad Católica de Chile, Santiago, Chile; ^2^Millennium Institute on Immunology and Immunotherapy IMII, Santiago, Chile; ^3^Chemical Biology and Drug Discovery Lab, Facultad de Medicina y Ciencia, Universidad San Sebastián, Santiago, Chile; ^4^Laboratorio de Endocrinología-Inmunología, Facultad de Ciencias de la Vida, Universidad Andres Bello, Santiago, Chile; ^5^Departamento de Genética Molecular y Microbiología, Facultad de Ciencias Biológicas, Pontificia Universidad Católica de Chile, Santiago, Chile; ^6^Department of Physiology, Facultad de Ciencias Biológicas, Pontificia Universidad Católica de Chile, Santiago, Chile; ^7^Center of Translational Endocrinology (CETREN), Department of Endocrinology, School of Medicine, Pontificia Universidad Católica de Chile, Santiago, Chile

**Keywords:** obesity, aldosterone, mineralocorticoid receptor, MR antagonists, eplerenone, high-fat diet animal model, RAAS

## Abstract

**Introduction:** Mineralocorticoid receptor (MR) activation within adipose tissue, triggers inflammation and metabolic syndrome development. The pharmacological blockade of MR provides beneficial effects for adipose tissue. Our study evaluates the impact of eplerenone implantation upon obesity.

**Experimental approach:** A group of mice with implanted placebo pellets were fed using two types of diet, a normal (ND) or a high fat (HFD) diet. Additionally, a group of mice fed HFD were implanted with an eplerenone pellet. Metabolic and biochemical parameters were assessed in each animal group. Adipocyte size and lipid accumulation were investigated in the liver and adipose tissue. We evaluated the components of renin-angiotensin-aldosterone system (RAAS) locally in adipose tissue.

**Key results:** Eplerenone reduced HFD-induced body weight gain, fasting glucose levels, fat accumulation, HFD-induced adipocyte size and liver lipid accumulation and improved glucose tolerance. In the adipose tissue, HFD significantly increased the mRNA levels of the RAAS molecules relative to the ND group. Eplerenone lowered RAAS mRNA levels, components of lipid metabolism and markers of inflammation in HFD-fed animals.

**Conclusion:** MR antagonism with eplerenone diminishes insulin resistance that is related to obesity partly via a reduction of RAAS activation, inflammatory progression and cytokines induction. This suggests that eplerenone should be further studied as a therapeutic option for obesity and overweight.

## Introduction

Obesity is currently, considered a pandemic and is associated with overexpansion of fatty tissue, insulin resistance and metabolic disorders namely increased blood glucose (diabetes), blood lipid levels and blood pressure (hypertension) (1, 2). Obesity is characterized by having a chronic and systemic low-grade inflammation, which arises partially from adipose tissue inflammation caused by the presence of immune cells, like macrophages and T cells, and secretion of proinflammatory substances that spill into the bloodstream, thus contributing to systemic inflammation (3). The dysfunction of adipocytes and adipose tissue may cause health problems such as obesity and metabolic syndrome (MetS) (4). The excess of adipose tissue frequently contributes to endocrine modifications and other tissues dysfunction such as liver and skeletal muscle (5).

Preclinical studies have demonstrated that activation of MR works on adipocyte function, resulting in significant changes in biochemical and morphological markers of adipose tissue differentiation (6, 7) positioning MR as a fundamental proadipogenic transcription factor modulating the effects of aldosterone and glucocorticoids on adipose tissue and its pathophysiological role in obesity and the metabolic syndrome (8, 9). Significantly, analysis of terminally differentiated 3T3-L1 adipocytes treated with aldosterone and a MR antagonist, demonstrated that the MR modulates the expression of proinflammatory adipokines (*Tnfa, IL6*, and *Mcp1*), peroxisome proliferator-activated receptor gamma *(PPARg)*, adiponectin and enzymes involved in reactive oxygen species (ROS) production, suggesting that MR function has a fundamental role in modulating the activity of mature adipocytes. Furthermore, MR blockade has also been shown to reduce ROS levels and inflammation in mature adipocytes (6, 10).

Urbanet et al. demonstrated that development of MetS in mice can be explained by an increased MR expression in the adipose tissue of mice which results in various metabolic dysfunctions, comprising body weight gain, visceral obesity, glucose intolerance, insulin resistance, and dyslipidemia (11). Furthermore, both Caprio's group and others have shown that pharmacological MR blockade in mice implies beneficial outcomes on adipose tissue and glucose tolerance, thus combating obesity (12, 13). Likewise, other studies have proved in an obese mice model that the improvement of glucose metabolism is obtained after MR antagonism via modified expression of adipogenic and inflammatory markers in adipose tissue (12, 14–16). Furthermore, Wada et al. have recently demonstrated that MR inhibition in mice using eplerenone improves glucose intolerance and insulin resistance and prevents weight gain and fat accumulation being one of the mechanisms the inhibition of inflamasome mediated chronic inflammation (17). In humans, eplerenone has been used alone or in combination with other drugs to treat high blood pressure. Eplerenone binds to MR and blocks the binding of aldosterone lowering blood pressure (18).

Albeit the connection between obesity, MetS and insulin resistance has substantially been recognized (19–21), the mechanisms linking these complex diseases have yet to be determined. The renin-angiotensin-aldosterone-system (RAAS) has an identified role on regulating blood pressure, fluid, and electrolyte homeostasis (22). RAAS components within adipose tissue were identified in the late 1980s, and were conserved in human and rodent adipose tissue also in cultured adipocytes (23–30). Since RAAS presents autonomous regulation, the adipose tissue RAAS has issued not only as a pivotal modulator of adipose tissue metabolism but also of glucose, blood pressure and whole body energy homeostasis (20, 31, 32).

Herein, our aim was to study the impact of a subcutaneous pellet administration of the MR-antagonist eplerenone, on the systemic and epididymal White adipose tissue (eWAT) RAAS components and inflammation in an animal model of obesity.

## Materials and Methods

### Ethics Statement

Animal studies are reported in compliance with the ARRIVE guidelines (33, 34). All experimental protocols were reviewed and approved by the Scientific Ethical Committee for Animal and Environment Care of the Pontificia Universidad Católica de Chile (ID Protocol No. 170811001/2018). This agreement defined appropriate endpoints, which limited the amount of pain an animal suffered during the development of obesity. Animal behavioral changes were assessed on a daily basis.

### Animal Maintenance and Experimental Design

Animals were housed at the animal facility of the Faculty of Biological Sciences at Pontificia Universidad Católica de Chile under a 12-h light/12-h dark-cycle, at a temperature of 23°C in a humidity-controlled environment. The experimental protocols were carried out after a 1-week acclimation period within the animal care facility where mice were allowed *ad libitum* access to fluid and food [normal chow D12450B, Research Diets, New Brunswick, NJ, 10% fat, referred to as the normal diet **(ND)**]. This protocol was designed according to the ARRIVE guidelines (33). Seven-week-old male C57BL/6J mice from Jackson Laboratories (USA) were weighed and housed randomly in 3 groups with four mice per cage (20 mice per group). Mice were implanted with a subcutaneous pellet. Animals with placebo pellets were fed ND or a high-fat diet [NC-111 Placebo for Eplerenone (60 mg/pellet) 90-day release, referred to as the ND or HFD respectively]. To test the effects of MR antagonism, an additional high-fat diet group was implanted with a subcutaneous eplerenone pellet [Innovative Research of America (Sarasota, FL, USA)-AKSci (AK Scientific, Inc., NX-999 Eplerenone (60 mg/pellet) 90-day release, referred to as HFD-E)]. All animals were fed for 90 days. Body weight was measured twice a week. There is no literature showing the usage of sub dermic pellets of eplerenone in rodents, the choice of our dosage was similar than those chosen on previous studies where the eplerenone was added to chow (200 mg/kg pellet) (17, 35, 36). In our opinion the advantage of using pellets is that they deliver a constant and known dose of the drug Eplerenone to all animals regardless of the amount of food that each animal consumes. We used AK Scientific, Inc., NX-999 Eplerenone (60 mg/pellet) 90-day release, which delivers 0.67 mg/day of the drug. On the other hand, the protocols used by Lowea et al. (37), they expect that the animals consume an Eplerenone estimated dose of 200 mg/kg × day which corresponds to 0.42 mg/day. Thus, in an animal model of diet-induced obesity, the administration of eplerenone via a subcutaneous pellet to modulate adipocyte dysfunction might be a suitable strategy.

### Glucose Tolerance Tests (GTTs) and Insulin Tolerance Tests (ITTs)

Twelve-week-old mice were deprived of food for 6 h. For the GTTs, mice (20 per group) were intraperitoneally (i.p.) injected with glucose (glucose 1 g/kg bodyweight, Sigma-Aldrich, Shanghai, China). For the ITTs, mice were intraperitoneally injected with recombinant bovine insulin (1 U/kg body weight, Sigma- Aldrich, Shanghai, China). For each test, blood from the tail vein was collected 0, 30, 60, and 120 min after injection and the glucose level was measured using an automatic blood glucose meter [Accu-Chek- Performa Nano (Roche, Tokyo, Japan)].

### Analysis of Body Fat Composition by Magnetic Resonance Imaging (MRI)

To measure body fat mass and lean mass, mice were placed into the Echo MRI analysis system (4 in 1 Echo MRI-900TM; Echo Medical System, Houston, TX) as described previously (38). The mice were sacrificed, and tissues were collected for primary cell culture or paraffin sections or were stored at −80°C.

### Histological Analysis

Histological staining was performed as previously described (30). Briefly, isolated epididymal white adipose tissue (eWAT) and livers were fixed in 4% formalin solution at room temperature after sacrifice, embedded in paraffin, dewaxed, hydrated, and cut serially into 5-μm-thick sections (Leica RM2255, Shanghai, China). To assess the average diameters, numbers, and population of adipocytes, slides were stained with hematoxylin-eosin [H&E (#MHS16, Sigma-Aldrich)], and each slide image was evaluated in 5 random fields of 12 animals per condition using ImageJ Pro, Plus Version 6.0 (Media Cybernetics, Rockville, MA, USA). All assays were performed in a blinded manner, and the data are presented as the average cell diameter. Lipid droplets were stained with Oil Red-O (ORO, Sigma, Germany). Briefly, OCT embedded cells were stained with a working solution of ORO for 30 min at room temperature and rinsed three times with deionized water before microscope evaluation.

### Cell Culture

SW-872 (ATCC HTB-92) cells were seeded on plastic culture dishes (Nunc, Rochester, NY) and grown in DMEM/Ham's F-12 (1:1) medium (Sigma, St. Louis, MO) supplemented with 10% fetal bovine serum (FBS) and antibiotics (penicillin-streptomycin 100X, LIFE TECHNOLOGIES CHILE SPA) at 37°C in a controlled atmosphere incubator (5% CO_2_) until reaching a confluence of 70%.

### Cell Cycle Study

The growing cells were subjected to starvation for 16 h to allow synchronization (basal condition), before stimulation with the reported pharmacological treatments or their mixtures for a further 24 h. Cells were trypsinized, fixed in 6.6% ethanol, washed twice with PBS, counted and stained with 20, μg/mL propidium iodide solution (P4864-10 ml, Sigma-Aldrich, Chile) containing 5 μg/ml RNase A for 30 min at 37°C. Cell cycle analysis was performed using FACS Canto II cytometer (BD Bioscience) and FlowJo software for flow cytometry analysis. Pharmacological treatment: aldosterone 0.1 nM (A9477, Sigma), eplerenone 2.5 mM (E6657, Sigma-Aldrich, Chile) added 30 min prior to aldosterone challenge, dexamethasone 150 nM (D1756, Sigma), cortisol 150 nM (H4001, Sigma). Cell viability was controlled by MTS according to the manufacturer s instructions (Promega, Cell titer 96, one solution, #G3582).

### Serum Aldosterone and Renin Quantification

Aldosterone and renin levels were measured in plasma samples with commercial competitive ELISA kits according to the manufacturer's recommendations (EIAALD and EMREN1, respectively, Invitrogen, Thermo Fisher Sci., Frederick, MD, USA). The coefficient of variance of the intra-assay variability for the aldosterone RIA was 7.5%, and for the renin RIA was 6.3%.

### RNA Extraction and Real-Time PCR Analysis

Total RNA was purified from epididymal white AT (eWAT) via TRIzol (Invitrogen) extraction following the manufacturer's instructions. First-strand complementary DNA was synthesized using the Superscript First-Strand Synthesis kit (Invitrogen). Quantitative real-time PCR was performed using the Power SYBR GreenPCR Master Mix (Applied Biosystems) on RotorGene-6000 instrument (Corbett Research, Sydney, Australia) with reaction volumes of 20 μl. The delta-delta CT method was used to determine mRNA levels. Target gene expression was normalized to 18S rRNA mRNA levels (39, 40). Primer sequences are listed in Supplementary Table 1.

### Flow Cytometry Analysis

The stromal visceral fat fraction (SVF) and spleen were used for the analysis of major histocompatibility complex class II (MHCII) cells. For this purpose, the obtained fraction was stained with anti-MHCII-PercpCy5.5 (562363 BD), anti-CD11C-PE (117308 BioLegend), anti-CD80-FITC (553768 BD), and anti-CD11b-APC (553312 BD); washed; and resuspended in PBS-5% FBS. The samples were analyzed using a FACSCanto II analyzer (BD Bioscience). Data were analyzed using FlowJo software for flow cytometry analysis.

### Statistics Analysis

All data are presented as the means ± SEMs. Statistical analysis of weight gain, plasma glucose curves (GTT or ITT), and liver lipids, were performed were analyzed using Kruskal-Wallis and Dunn's tests. Plasma glucose area under curve (AUC) (GTT or ITT) and fasting plasma glucose using 2 way ANOVA and Tukey's tests. Total percentage of lean or percentage of fat by magnetic resonance imaging (MRI), cell cycle phase distribution or eWAT area were analyzed by one-way ANOVA followed by the multiple comparisons Tukey's test. The final weight gain %, aldosterone plasma levels and the aldosterone-renin ratio (ARR) were analyzed by Kruskal-Wallis and Dunn's tests. The normalized expression (2e– delta-delta CT method) was employed to calculate the normalized expression levels of each mRNA gene for each simple. All of the data were analyzed using Kruskal-Wallis and Dunn's tests. For pairwise comparisons, Student's *t-*test was employed. Correlation analyses were carried out using Pearson's product-moment correlation test. *P* < 0.05 indicated a significant difference. All analyses were performed using GraphPad Prism 8 software.

## Results

### High-Fat Diet (HFD)-Induced Obesity and Eplerenone Attenuated the Effect of HFD on Weight Gain and Glucose Tolerance

**Figure 1:**
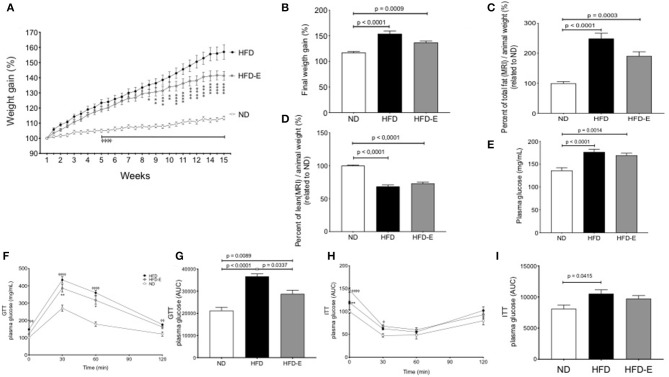
Effects of eplerenone on body weight gain, body fat composition and glucose metabolism in mice. Three groups of mice (normal diet-fed mice, ND: white; high-fat diet-fed mice, HFD: black; HFD with implanted eplerenone-pellet mice, HFD-E: gray). **(A)** Body weight gain over 15 weeks (*n* = 20 per group) ⌽, *p* < 0.001 ND vs. HFD or HFD-E; *p < 0.001 HFD vs. HFD-E. **(B)** Final weight after 13 weeks of diet treatments. **(C)** Quantitation of total fat mass is shown (*n* = 12–15 per group). **(D)** Quantitation of total lean mass is shown (*n* = 12–15 per group). **(E)** Final fasting glucose is presented as the mean ± SEM of glucose (mg/mL). **(F)** GTT (1 g of glucose/kg body weight, i.p.) was conducted in 6 h-fasted mice, and the results are expressed as the mean percentage of initial glucose at *t* = 0. ⌽, *p* < 0.05 ND vs. HFD; *p < 0.05 HFD vs. HFD-E. **(G)** The mean glucose area under the curve (AUC) over the course of 120 min in each group is shown (*n* = 12–15 per group). **(H)** ITT (1 unit of insulin/kg body weight, i.p.) was conducted in 4 h-fasted mice. **(I)** The averaged AUC over the course of 120 min for each group is shown (*n* = 7–8 per group). Data are shown as the mean ± SEM ⌽, *p* < 0.05 ND vs. HFD; *p < 0.05 HFD vs. HFD-E. Graphs **(B,C,D,E,G,H,I)** were analyzed using Kruskal-Wallis and Dunn's tests; **(A,F,H)** via 2 way ANOVA and Tukey's tests. *p < 0.05; **p < 0.01; ***p < 0.001; ****p < 0.0001.

C57BL/6 mice fed a ND, HFD or HFD-E showed different profiles of body weight changes during the experimental period. Both HFD and HFD-E mice showed a linear and continual increase in body weight and were significantly different from that of the ND mice from week 5 onward. The body weight gain of the HFD-E mice was significantly different from that of the HFD mice from week 8 onward (Figure 1A). The final body weight was significantly higher in the HFD group than that in the ND (31.3% increase *p* < 0.0001) or HFD-E (16.6% increase, *p* = 0.009) groups. No significant modification of food intake or initial weight occurred (Supplementary Figure 1). Eplerenone treatment attenuated body weight gain after 15 weeks in mice fed with HFD compared to HFD animals (a 47% decrease) (Figure 1B). Evaluating the body composition by MRI, the HFD group exhibited significantly more fat tissue content than that of the ND (128% increase, *p* < 0.0001) and HFD-E (76% increase, *p* = 0.0003) groups. Comparing HFD vs. HFD-E animal we observed a 48% reduction of fat mass although without statistical significance (Figure 1C). Upon examination of lean mass, the HFD and HFD-E group had a significantly lower lean mass than the ND group (*p* < 0.00001 and *p* < 0.0001, respectively). The lean mass in the eplerenone-treated group was slightly higher than HFD group but without statistical significance (Figure 1D).

### Glucose Tolerance Tests (GTTs) and Insulin Tolerance Tests (ITTs)

Fasting glucose levels differed between each group; the levels in the HFD group were significantly higher than those in the ND (*p* < 0.0001; Figure 1E). GTT was impaired in HFD group compared to the findings in the ND (*p* < 0.0001) and this effect was reversed by eplerenone in the HFD-E group (*p* = 0.0337; Figures 1F,G). Similar results were found in response to glucose levels during the ITT, although HFD mice did not differ from HFD-E mice (Figures 1H,I).

### Effects of Eplerenone on Obesity-Induced Histological Changes in Epididymal White Adipose Tissue (eWAT) and Liver

**Figure 2:**
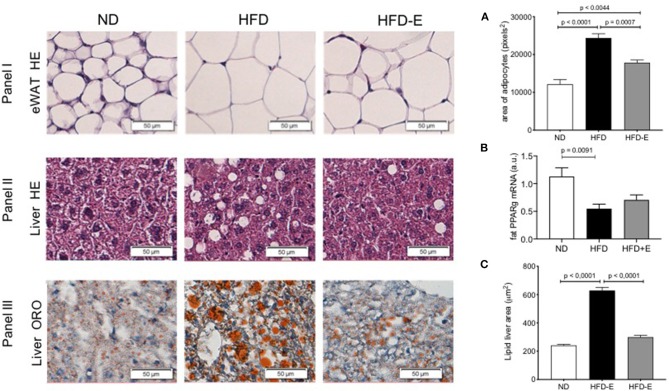
The effects of eplerenone on the morphological characteristics were examined by histological analysis of the eWAT and liver of mice. Three groups of mice (normal diet-fed mice, ND: white bar; high-fat diet-fed mice, HFD: black bar; HFD + eplerenone mice, HFD-E: gray bar) were analyzed. Representative photomicrograph of hematoxylin and eosin (HE)-stained sections of eWAT are shown (upper panel). **(A)** Average size of epididymal white adipocytes (eWAT). Scale bar, 50 μm. Representative photomicrograph of hematoxylin and eosin (HE)-stained sections of the liver (middle panel). Scale bar, 50 μm. Representative photomicrograph of ORO-stained sections of the liver (lower panel). Scale bar, 50 μm. **(B)** PPARgamma mRNA expression in the eWAT. **(C)** Triglyceride content in the liver. Data are shown as the mean ± SEM (*n* = 12). The sizes of eWAT and lipid droplets in the liver were measured with ImageJ. The area of over 200 adipocytes was measured for each tissue sample. Data are presented as the mean ± SEM. Graphs were analyzed using Kruskal-Wallis and Dunn's tests.

To evaluate eWAT (Figure 2, panel I), liver morphology (Figure 2, panels II and III), and lipid storage induced by HFD-feeding and the effect of eplerenone treatment, we conducted HE (Figure 2, panels I and II) and Oil-Red O staining (Figure 2, panel III). Adipocyte size markedly increased upon HFD feeding relative to the ND group (*p* < 0.0001) and this effect was lost in the HFD-E group (*p* = 0.0044; Figure 2A), thus eplerenone treatment significantly suppressed adipocyte hyperplasia (*p* = 0.0007). When analyzing the expression of PPARgamma in these tissues we found that HFD significantly decreases the expression in relation to ND (*p* = 0.0091), however treatment with HFD-E was not different from ND (Figure 2B). Analysis of liver fat content showed statistically significant results as those found in adipocytes. Lipid accumulation in the hepatic lobules in HFD mice was markedly increased by HFD feeding (*p* < 0.0001) and reduced in HFD-E mice (*p* < 0.0001; Figure 2C).

### Effects of Eplerenone on the Adipocyte Cell Cycle

Considering the results obtained after the treatment with eplerenone on adipose tissue and the size and number of adipocytes, we hypothesized that maybe eplerenone has an effect on cell cycle. For this reason, we studied the phases of the cell cycle of preadipocytes using the SW-872 cell line through an *in vitro* assay. The basal or control condition arrests the growth of cells after 24 h of preadipocyte cell starvation (DMEM- F12+1% FBS without steroids). The percentage of change in the arrest cycle cells phases related to growing conditions (10% FBS with steroids) were, a significant decrease in those that are proliferating (S+G2M) (*p* = 0.0159) and a significant increase in cells in those that are in G1 (*p* = 0.0034). The commitment genes increase their mRNA expression according with the changes in cell cycle phases (Supplementary Figure 2A). Taking this basal point, we stimulated with 0.1 nM aldosterone, 2.5 μM eplerenone, or a combination of both drugs for further 24 h. Aldosterone significantly increased the percentage of cells that were in (S+G2M) (19%, *p* = 0.0450) related to basal condition, this effect was prevented by the presence of eplerenone, which returned the status to basal conditions.

**Figure 3:**
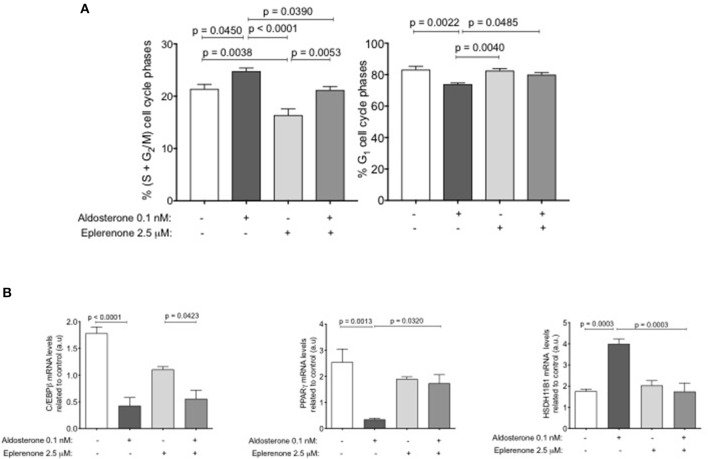
The effect of aldosterone or eplerenone on the adipose cell cycle. **(A)** Percent of cells in S+G_2_M (left) or G_1_ (right) phases. White bar, basal condition after starving; dark gray bar, aldosterone (0.1 nM) clear gray, aldosterone + eplerenone (2.5 μM) antagonist, middle gray bar only eplerenone treatment. **(B)** mRNA levels of the early PPARgamma, C/EBPbeta and middle HSD11B1 adipogenic genes related to basal conditions (same colors bars as for treatments for the cell cycle). Data are presented as the mean ± SEM. *P* < 0.05 was considered statistically significant. Graphs were analyzed using Kruskal-Wallis and Dunn's tests.

Treatment with the eplerenone alone, significantly reduced the cells in (S+G2M) (24%, *p* = 0.0038) compared to that in the basal condition, indicating a basal MR effect on cell cycle proliferation. The inverse was found for the cells that were in G1, where aldosterone significantly decreased the percentage of cells that were in G1 (*p* = 0.0022). When cells were stimulated with eplerenone or in combination with aldosterone, we found no difference in the percentage of cells in G1 in relation to the basal condition (Figure 3A). To study if these effects were specific of aldosterone on MR, we study the effect of 150 nM dexamethasone and/or 150 nM cortisol (GR agonists) on cell cycle phases. Neither dexamethasone (black S+G2M bar) nor cortisol (gray S+G2M bar) reduced the percentage of cells in the proliferative phase (Supplementary Figure 2B), on the contrary, they increased this percentage. No concentration of any drug used, affected cell viability (Supplementary Figure 2C). In parallel, using the same conditions, we examined the expression levels of early and intermediate commitment genes in these adipocytes (Figure 3B).

Aldosterone addition resulted in a significant decrease in the expression of C/EBPbeta and PPARgamma mRNAs (*p* < 0.0001; *p* = 0.0013, respectively) in relation to the basal conditions. Eplerenone presence partially reversed the effect of aldosterone, slightly increasing the expression of C/EBPbeta mRNA. In relation to PPARgamma mRNA, eplerenone reversed the effect of aldosterone (*p* = 0.0320). For HSD11B1, we found the opposite effect, aldosterone significantly increased the expression of its mRNA (*p* = 0.0003), and the combination of drugs reversed that increase.

### Both, the Local and Circulating Adipose Tissue Renin-Angiotensin–Aldosterone System (RAAS) Is Activated in HFD Mice

**Figure 4:**
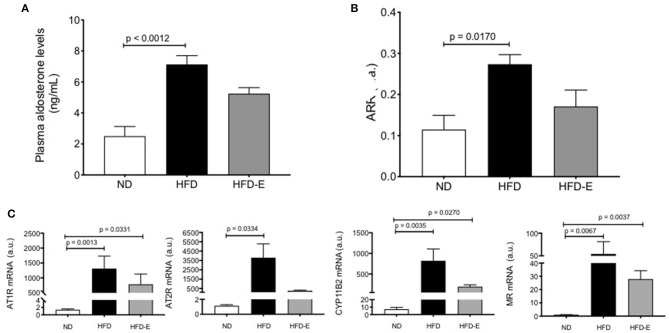
Effects of eplerenone on aldosterone plasma levels and RAAS molecule expression in eWAT. Three groups of mice (normal diet-fed mice, ND: white bar; high-fat diet-fed mice, HFD: black bar; HFD + eplerenone mice, HFD-E: gray bar) were analyzed. Plasma aldosterone levels **(A)** and the aldosterone-renin ratio **(B)**. mRNA expression levels of AT1R, AT2R, CYP11B2, and mineralocorticoid receptor (MR) genes in the eWAT of each group are shown **(C)**. Data are represented as the mean ± SEM. *P* < 0.05 was considered significant. Graphs **(A–C)** were analyzed using Kruskal-Wallis and Dunn's tests.

Plasma levels of aldosterone were significantly increased in HFD mice when compared to the ND (*p* = 0.0012) indicating that this higher concentration can potentiate the RAAS components in the mouse adipose tissue (HFD: 7.2 ± 0.8 ng/ml, *n* = 8 vs. ND: 2.6 ± 0.5 ng/ml, *n* = 8, *p* = 0.0012; Figure 4A). Similar results were observed with the angiotensin renin ratio (ARR) (Figure 4B). To assess the effect of HFD on local adipose tissue, we evaluated the mRNA expression levels of some RAAS system components on eWAT (Figure 4C), thus AT1R (*p* =0.0013, *n* = 9), AT2R (*p* = 0.0334, *n* = 9), CYP11B2 (*p* = 0.0035, *n* = 9), and MR (*p* = 0.0067, *n* = 9) were increased in the HFD mice (Figure 4, lower panel).

### Relationship Between Circulating RAAS and Fat Mass

**Figure 5:**
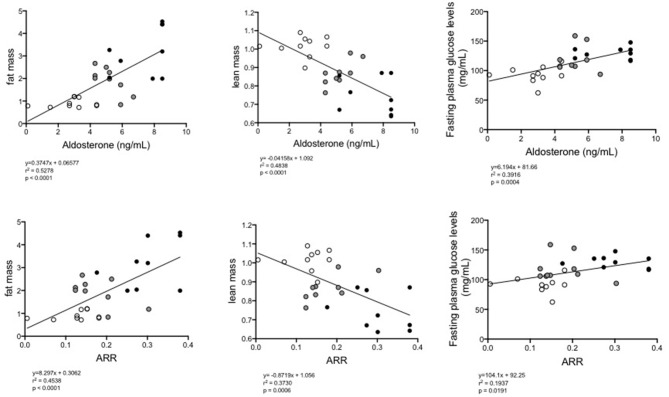
Linear regression between plasma levels of aldosterone (upper) or the ARR (lower) and fat% (left), lean mass% (middle), or fasting glucose levels (right) of all groups of mice (normal diet-fed mice, ND clear circles: high-fat diet-fed mice, HFD black circles: HFD + eplerenone mice, HFD-E gray circles). *P* < 0.05, was considered significant.

In view of the previous results, we wanted to determine if there was an association between aldosterone plasma levels and the aldosterone-renin ratio (ARR) and metabolic variables, such as the percentage of fat, lean mass content and glucose levels, as well as the local expression of the RAAS components in eWAT. Using Spearman correlation analysis, a positive correlation was observed between the concentration of aldosterone (*p* = 0.0028, rho = 0.5431) and the ARR (*p* = 0.05, rho = 0.3634) and the percentage of fat tissue measured by MRI (Table 1). A negative correlation was observed between the concentration of aldosterone (*p* < 0.0001, rho = −0.6955), the ARR (*p* = 0.0006, rho = −0.6108) and lean mass. With respect to blood glucose levels, a positive correlation was observed with the increasing of aldosterone levels (*p* = 0.0004, rho = 0.6258) and the ARR (*p* = 0.0191, rho = 0.4402). The increase in plasma aldosterone levels was positively associated with the expression of CYP11B2 (*p* = 0.0005; rho = 0.6961), AT1R (*p* = 0.0030; rho = 0.6281), and AT2R (*p* < 0.0001; rho = 0.8434) mRNAs. There was a positive association between ARR and CYP11B2 (*p* = 0.0387; rho = 0.4540) and AT2R (*p* = 0.0002; rho = 0.7066). When the complete group of mice was analyzed as a whole regarding the continuum of cardiometabolic risk, linear regression between the concentration of aldosterone or the ARR and the fat mass percentage (*y* = 0.3747X + 0.06577, and *y* = 8.297X + 0.3062, respectively) or plasma glucose levels (*y* = 6.194X + 0.81.66, and *y* = 104.1X + 0.0191, respectively) revealed a directly proportional relationship, and the inverse was observed for the lean mass percentage (*y* = −0.04158X + 1.092 and y = −0.8719X + 1.056, respectively; Figure 5).

**Table 1:** Relation between circulating renin-angiotensin–aldosterone system and fat and lean mass, fasting glucose levels, and expression of CYPB11B2, AT1R, and AT2R.

	**Aldosterone ng/ml**		**ARR (a.u.)**	
	***p***	***R***	***p***	***R***
Fat mass percentage	0.0028	0.5431	0.05	0.3634
Lean mass percentage	<0.0001	−0.6955	0.0006	−0.6108
Fasten glucose mg/ml	0.0004	0.6258	0.0191	0.4402
CYP11B2 mRNA expression	0.0005	0.6961	0.0387	0.4540
AT1R mRNA expression	0.0030	0.6281	0.0900	0.3890
AT2R mRNA expression	<0.0001	0.8434	0.0002	0.7066

*Fat, lean mass%, and glucose n = 20; mRNA n = 7*.

### Effects of Eplerenone on Proinflammatory Cytokines Gene Expression and Adipose Metabolism Component Molecules in Epididymal White Adipose Tissue (eWAT)

**Figure 6:**
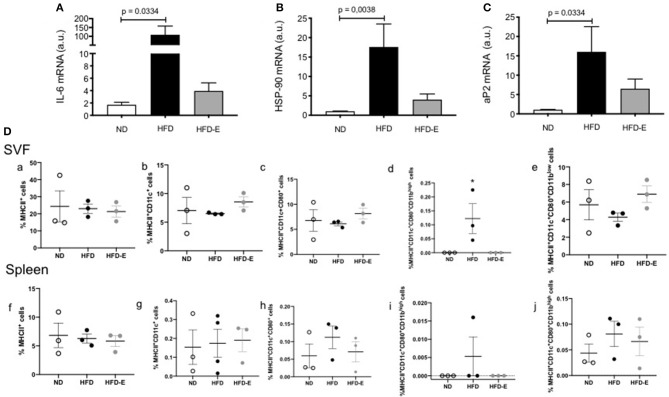
Upper panel: Effects of eplerenone on the mRNA expression levels of metabolic and inflammatory molecule genes in the eWAT **(A–C)**. Three groups of mice (normal diet-fed mice, ND: white bar; high-fat diet-fed mice, HFD: black bar; HFD + eplerenone mice, HFD-E: gray bar) were analyzed. Data are presented as the mean ± SEM. *P* < 0.05 was considered significant. Graphs were analyzed using Kruskal-Wallis and Dunn's tests. Lower panels: Eplerenone treatment decreased the percentage of MCHII^+^CD11b^high^ cells in the SVF. The percentage of MCHII^+^ **(a,f)**, MHCII^+^CD11c^+^ **(b,g)**, MHCII^+^CD11c^+^CD80^+^ **(c,h)**, MHCII^+^CD11c^+^CD80^+^ CD11b^high^ **(d,i)**, and MHCII^+^CD11c^+^CD80^+^CD11b^low^ **(e,j)** was analyzed in the SVF and spleen of ND, HFD, and HFD-E mice. The obtained results showed an increase in MHCII^+^CD11c^+^CD80^+^ CD11b^high^ in the HFD mice that was reversed by eplerenone treatment. Mean ± SEM. *N* = 3, ANOVA and Tukey's *post-hoc* tests. Graph **(D)** was analyzed via one way ANOVA test.

To study the effects of eplerenone on chronic inflammation associated to obesity, we measured the mRNA levels of IL6, HSP90, and aP2. In eWAT, the expression levels of IL6 (*p* = 0.0334), HSP90 (*p* = 0.0038), and aP2 (*p* = 0.0334) were significantly increased in HFD mice relative to ND mice. While the expression levels of these molecules were attenuated in the presence of eplerenone, they did not reach significant differences from HFD group (Figures 6A–C). In addition, in a small sample of animals (*n* = 3), we analyzed the selected immune populations in the stromal visceral fat fraction (SVF) and the spleen of the ND, HFD, and HFD-E mice groups by flow cytometry. The statistical analysis did not show significant differences between the groups when the total MHCII + cells were analyzed. Interestingly, when the subpopulations of MCHII + cells were analyzed, a significant increase in MHCII + CD11c + CD80 + CD11 was observed in the SVF of the HFD mice (*p* = 0.05), while treatment with eplerenone reduced the percentage of these cells, which restored the phenotype to that of the control (Figure 6D).

## Discussion

Herein, we present an animal model demonstrating that RAAS activation occurs in animals whose body weight is increased through HFD feeding. Eplerenone reduced the increase in body weight, improved glucose metabolism, modified the RAAS components and reduced adipocyte size and lipid accumulation in the liver, suggesting that MR antagonists might be candidates to prevent aspects of metabolic disorders under obese conditions. Our results agree with those published by Wada et al. relating the effects of eplerenone on glucose metabolism and reduced body weight and fat accumulation in mice. They also found that antagonizing MR by eplerenone prevented the activation of the inflammasome via inhibition of two components Nlrp3 and Caspase1 (17).

The novelty of our results lies in determining what is the role of eplerenone on the RAAS components in adipocytes, an issue that has not been addressed so far. Our results together with those of Wada et al., allow us to have a broader vision of how eplerenone is acting at the cellular and molecular level. Therefore, it is very important to demonstrate that these results are extensible to humans, which would allow us to have another tool to treat obesity and associated morbidities. We found that HFD significantly decreases PPARgamma when compared to ND, and the addition of eplerenone reverses this effect although not statistically significant (*p* = 0.091 HFD vs. ND; not significant HFD-E vs. ND), similar to what was found *in vitro* (SW-872 supplemented with aldosterone would correspond to animals fed with HFD).

In our model we have not measured the aldosterone levels both in cell culture or eWAT tissue itself, we do not know how much aldosterone is being liberated from the cells even though we are able to see an increase on expression of aldosterone synthesis genes and plasma levels. One alternative would be to measure in cell culture the aldosterone liberation after induction using a very highly sensitive detection method (ELISA and or HPLC). Recently, Assersen et al. (41) have described that human perivascular adipose tissue is not able to secrete aldosterone nevertheless these authors do not demonstrate that CYP11B2 level, the enzyme responsible for aldosterone synthesis, is changed. No other study has demonstrated that human fat tissue secretes aldosterone. Undoubtedly, this is one question that has to be addressed in humans.

In this study we show that mice fed with HFD increased weight, fat percentage, decreased lean mass and had problems with glycemic control very similar to that observed in humans with MetS. HFD-fed mice also showed increased aldosterone plasma levels relative to that fed with ND or to which Eplerenone was added. In contrast, Chaudury et al. have published that HIV patients treated with eplerenone increase hepatic steatosis, a result that is not in agreement with ours in mice. Nevertheless, the sample studied corresponds to only five patients bearing multiple associated co-morbidities and the results not necessarily will be the same in healthy individuals (42). The association between aldosterone plasma levels and weight and fat accumulation has also been seen in humans, where the body mass index (BMI) has been shown to have a direct correlation with plasmatic concentration of aldosterone and excretion of aldosterone in the urine (43). Weight loss in obese patients is accompanied by decreased aldosterone levels, suggesting a connection between the production/secretion of aldosterone and the adipose tissue, a condition similar to that shown in mice fed with HFD but supplemented with eplerenone.

Studies of obese diabetic mice have shown that blocking the MR is able to decrease the adipocyte size and reduce the expression of prothrombotic and proinflammatory factors in adipose tissue, in conjunction with an increased expression of adiponectin, therefore potentially reversing the adipocyte dysfunction induced by the excess of fat tissue (6). In our model, histological changes in eWAT and liver morphology demonstrated that eplerenone could reduce the size of adipocytes and the accumulation of lipids in the liver of mice fed with HFD. In addition, the antagonism of MR may interrupt the vicious adipose metabolic cycle, leading to the suppression of adipocyte hypertrophy. Our *in vitro* studies showed that aldosterone induced a shift to the proliferative phase (S+G2M) of the preadipocyte population and that eplerenone was able to reverse the effect by modulating the expression of the key commitment markers C/EBPbeta, PPARgamma and HSD11B1, thereby preventing this effect. Similar findings were previously communicated, suggesting that aldosterone is able to mediate the very early initiation of adipocyte differentiation (44, 45).

By analyzing fasting glucose levels and the glucose test tolerance, our study demonstrated impaired glucose level management. Fasten glucose levels were high in HFD fed mice. The glucose tolerance test showed an improvement of glycemic indices upon eplerenone use. We also found a positive correlation between aldosterone or the ARR and the glucose levels. Hyperaldosteronism is associated with impaired carbohydrate metabolism has antecedently been granted to a defective secretion of insulin from pancreatic beta cells owing to hypokalemia, fibrosis, or a direct inhibitory outcome of corticosteroids on beta cells (46–48).

It has also been reported that patients with primary aldosteronism have an increased incidence of IR that it is mediated by a diminished expression of genes involved in lipid metabolism in visceral adipose tissue compared to that of sex, age, and BMI-matched controls (49). Moreover, aldosterone has different effects on insulin metabolism, among others, interferes with insulin signaling pathways, reduces insulin receptor gene transcription and enhances degradation of insulin receptor substrates and is able to increase oxidative stress and inflammation (50). Further grounds for a cause and effect dependence between hyperaldosteronism and metabolic syndrome is sustained analyzing the phenotype of a new transgenic mice model bearing a selective overexpression of MRs in adipocytes, which is characterized by insulin resistance and increased visceral fat mass, body weight and plasma triglycerides levels (51). “Karashima et al. analyzed adult patients with hyperaldosteronism but without abdominal obesity (52). The authors found that the effects of eplerenone on different variables—including body mass index and visceral adipose tissue—were reduced, as measured by computed tomography and fat scan, although the BMI of the participants was rather normal and suggested no redistribution of fat (52). On the contrary, in our study mice exhibited fat accumulation and high levels of aldosterone in relation to the control group. These high levels of aldosterone could not only act on MR but also on other receptors or they may allow for formation of heterodimers that modulate the expression of other genes.”

In relation to the change in mRNA expression levels of RAAS molecules we find an increase in these in mice fed with HFD. In obese subjects, adipocyte MR expression is high as well as, being higher in visceral adipose tissue than expression in subcutaneous adipose tissue (11). In our obesity model, mice fed with HFD not only showed an increase in circulating aldosterone but also a significant increase on the expression levels of the AT1R, AT2R, CYP11B2, and MR genes in adipose tissue than those in the ND group, an increase that was attenuated within mice in the presence of eplerenone. Regarding obesity, the activation of RAAS system has been shown both in human patients and rat adipose tissue (53, 54).

Moreover, obesity is associated with an upregulation of angiotensin-converting enzyme (ACE) in adipocytes (27, 55), and augmented levels of circulating angiotensin II (AngII) (56). On the contrary, when a group of obese women lose weight, plasma levels of aldosterone, angiotensinogen, renin, and plasma ACE activity are normalized, this event is also correlated with a reduction in blood pressure (57). Aldosterone, one of the essential components of the RAAS system has also a positive correlation with obesity (58, 59), suggesting a role between hypertension associated to obesity and enhanced mineralocorticoid levels. Apparently, local adipose RAAS would function independently of plasma RAAS through a proper control of renin levels but influencing circulating RAAS (60).

HFD increased the mRNA expression of the RAAS components (AT1R, AT2R, CYP11B2, and MR) within eWAT, as well as plasma levels of aldosterone. This increase was lowered in the presence of eplerenone. The reduction in plasma aldosterone levels and adipose RAAS component expression in HFD-E could be explained by the eplerenone-mediated reduction in adipocyte size and regulation of glycaemia plasma levels, which appears to interrupt the vicious adipose metabolic cycle. Leptin has been identified as a main regulator of CYP11B2 expression and aldosterone secretion in human and mice (61). Leptin produced by adipocyte can stimulate adrenal glands to release aldosterone, which promotes adipose differentiation activating MR. In our model, fat leptin mRNA expression increases in both HFD and HFD-E relative to ND animals, even though it was not statistically significant. In immunoblot analysis we did not find significant differences in adipose tissue. Perhaps in adipose tissue the expression of leptin receptors changes, which is pending for this study. Leptin levels in serum have not been measured either, but we assumed that they are elevated in animals fed with HFD.

Another phenomenon which has been described with the use of other MR-antagonists (spironolactone and drospirenone) is the browning of WAT, as evidenced by the increased protein and mRNA levels of the uncoupling protein-1 (UCP-1) within the e-WAT (13). The authors found that beige adipocytes emerged in visceral and inguinal fat of HFD-mice after treatment with MR antagonists, in association with a reduced rate of autophagy. In our study, immunoblot (using cox4 and tom20 as controls) and real time PCR (using gene 36B4 as controls) revealed a very low expression of UCP-1, which did not change with any treatment. Furthermore, the immunoblot for UCP-1 was negative (not shown).

To examine adipogenic function in obese mice, we analyzed the mRNA levels of IL6, HSP90, and aP2, finding them significantly higher in eWAT than in mice that were fed with ND. The addition of eplerenone attenuated the increased expression of these genes. It is recognized that adipose tissue produces both proinflammatory and anti-inflammatory adipokines. Thus, in obese subjects, there is a risen expression of proinflammatory cytokines, comprising TNFa, IL6, IL8, and MCP1 and the associated cardiovascular disease. It has been shown that MR activation and aldosterone increase the expression of proinflammatory adipokine in rodent adipose tissue, which has as a consequence a reduction in insulin receptor expression and impaired glucose uptake induced by insulin (62, 63). In fact, both mRNA and protein expression levels of insulin receptors are reduced in the subcutaneous adipose tissue from patients with primary aldosteronism (64).

It has been proposed that in obesity, dendritic cell (DC) particularly recruit the CD11b+ subset (65), and promote Th17 and Th2 cell responses, increasing their production of proinflammatory cytokines, such as IL6, IL1b, and IL23 (66, 67), which is consistent with the observation in the HFD mice where IL6 expression was significantly increased when compared to the expression associated with the control and HFD-E treatments. In this work, we also analyzed the DC population in the SVF of animals fed a ND, HFD and HFD-E diet. Using DC pan-markers, we were able to observe an increase in CD11c+CD11b+ DCs in the HFD mice compared to the levels in the ND or HFD-E mice. This phenotype corresponds to myeloid DCs and has been identified in peripheral tissues (68).

Eplerenone significantly reduced weight gain on HFD. Nevertheless, we cannot specifically quantify how the improvements in the metabolic parameters with eplerenone was mediated by the reduced weight gain because the different variables have different sensitivities. From our results, HFD increases 31.3% the body weight while HFD-E increases 16.6%, i.e., the eplerenone treatment by itself diminishes 50% of the body weight increase, which in terms of volume is a substantial reduction, but it was not statistically significant. Similar results were obtained with the content of fatty tissue. This means, that nearly a third of fat tissue was reduced via eplerenone, but this saving in fat accumulation was not statistically significant. On the other hand, the variable glycaemia was more sensitive to changes in diet. HFD increases glycaemia 80% and eplerenone significantly decreases the 50% of this increase. The adipocyte size was also a parameter sensitive to diet. Eplerenone supplementation decreases 50% the adipocyte size and 80% the liver fat deposit. Both the change in adipocyte size and glycaemia would be increasing an improvement on adipogenic dysfunction generated by the HFD. Even though the decrease in fat content is not statistically significant that does not mean that in physiological terms it does have important effects. The impact of sex is a limitation of our study and we have planned to do it in females shortly, thus using females could mean different results depending on their hormonal regulation. Therefore, our results cannot be generalized to the whole population because in our model we did not include female mice and we used young male mice starting the eplerenone treatment together with the HFD.

## Conclusion

In our mice model, MR blockade through subcutaneous administration of Eplerenone, prevented adipogenic dysfunction by improving the management of circulating glucose levels. These improvements in part would be through the reduction of RAAS activation, which prevented inflammatory progression and the induction of cytokines, suggesting the potential of eplerenone as a therapeutic option for obesity and overweight. Adverse adaptive mechanisms, such as overactivation of RAAS are recognized to contribute to obesity and heart failure. Therefore, when you want to prevent or delay its development, the anomalous activity of MR in non-cardiac comorbidities comes out as a likely effective target for new precision therapeutic approaches.

## Data Availability Statement

The datasets generated for this study are available on request to the corresponding author.

## Ethics Statement

This animal study was reviewed and approved by Scientific Ethical Committee for Animal and Environment Care of the Pontificia Universidad Católica de Chile (ID Protocol No. 170811001/2018).

## Author Contributions

AV and CAF contributed to the conception and design of the study. AV, CAF, NM-D, and MO performed the *in vitro* experiments. CAF and AV performed the *in vivo* experiments. AV, CL, GO, and CEF wrote the manuscript. All the authors contributed to the analysis and interpretation of data, and critically reviewed and approved the manuscript.

## Conflict of Interest

The authors declare that the research was conducted in the absence of any commercial or financial relationships that could be construed as a potential conflict of interest.

## Supplementary Material

Supplementary Figure 1 F-1S shows **(A)** the initial weights of the three groups studied (normal diet-fed mice, ND: white; high-fat diet-fed mice, HFD: black; HFD with implanted eplerenone-pellet mice, HFD-E: gray) and **(B)** Food intake. No significant differences were found between the groups.

Supplementary Figure 2 F-2S shows the **(A)** cells that are in (S+G_2_M) phase and cells in G_1_, phase before and after arrest by starving with 1% FBS without steroids (FBST). The 1% FBST treatment for 24 h changes the distribution of the cell cycle phases by decreasing cell replication from 40 to 20% (S+G2M) and increasing the arrest (G1) of the total from 60 to 80% of the cell population. Lower panel represents changes in mRNA expression of early and middle commitment genes, before and after arrest by starving with 1% FBS without steroids The mRNA levels of the early C/EBPbeta, PPARgamma or medium 11β-HSDH1 adipogenic genes increased according to the phases of the cell cycle (same cell cycle bar color). Data are presented as the mean ± SEM. *P* < 0.05 was considered significant. **(B)** Neither dexamethasone 150 nM (black) nor cortisol 150 nM (gray) reduced the percentage of cells in the proliferative phase, but on the contrary, they increased this percentage. **(C)** No concentration of drug used affected cell viability. Data are presented as the mean ± SD. Values under 2 standard deviations from the mean were considered toxic (*n* = 5 per bar).

Supplementary Table 1 List of primer sequences for measurement of tissue mRNAs.
