# Supplementary figures and images for: Eplerenone Implantation Improved Adipose Dysfunction Averting RAAS Activation and Cell Division
Source: Front Endocrinol (Lausanne). 2020 Apr 21;11:223. doi: 10.3389/fendo.2020.00223 (PMC7186315; doi:10.3389/fendo.2020.00223)

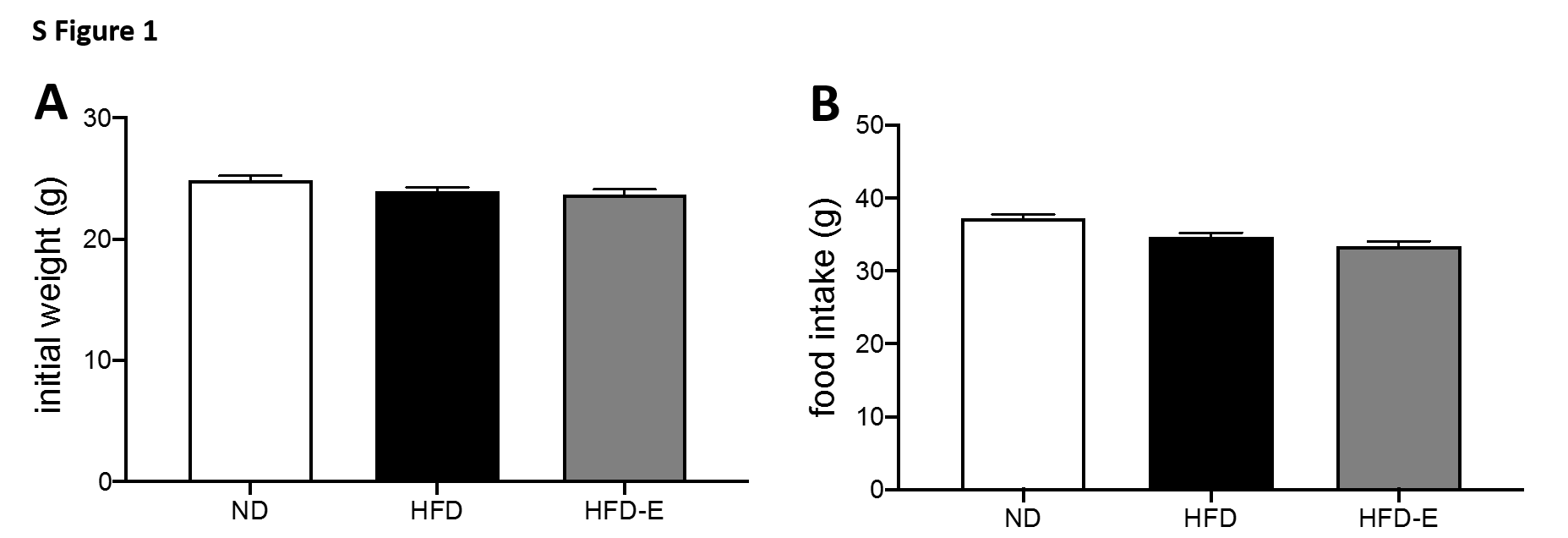

Supplement: Supplementary Figure 1 — F-1S shows (A) the initial weights of the three groups studied (normal diet-fed mice, ND: white; high-fat diet-fed mice, HFD: black; HFD with implanted eplerenone-pellet mice, HFD-E: gray) and (B) Food intake. No significant differences were found between the groups. [file Image_1.TIF]

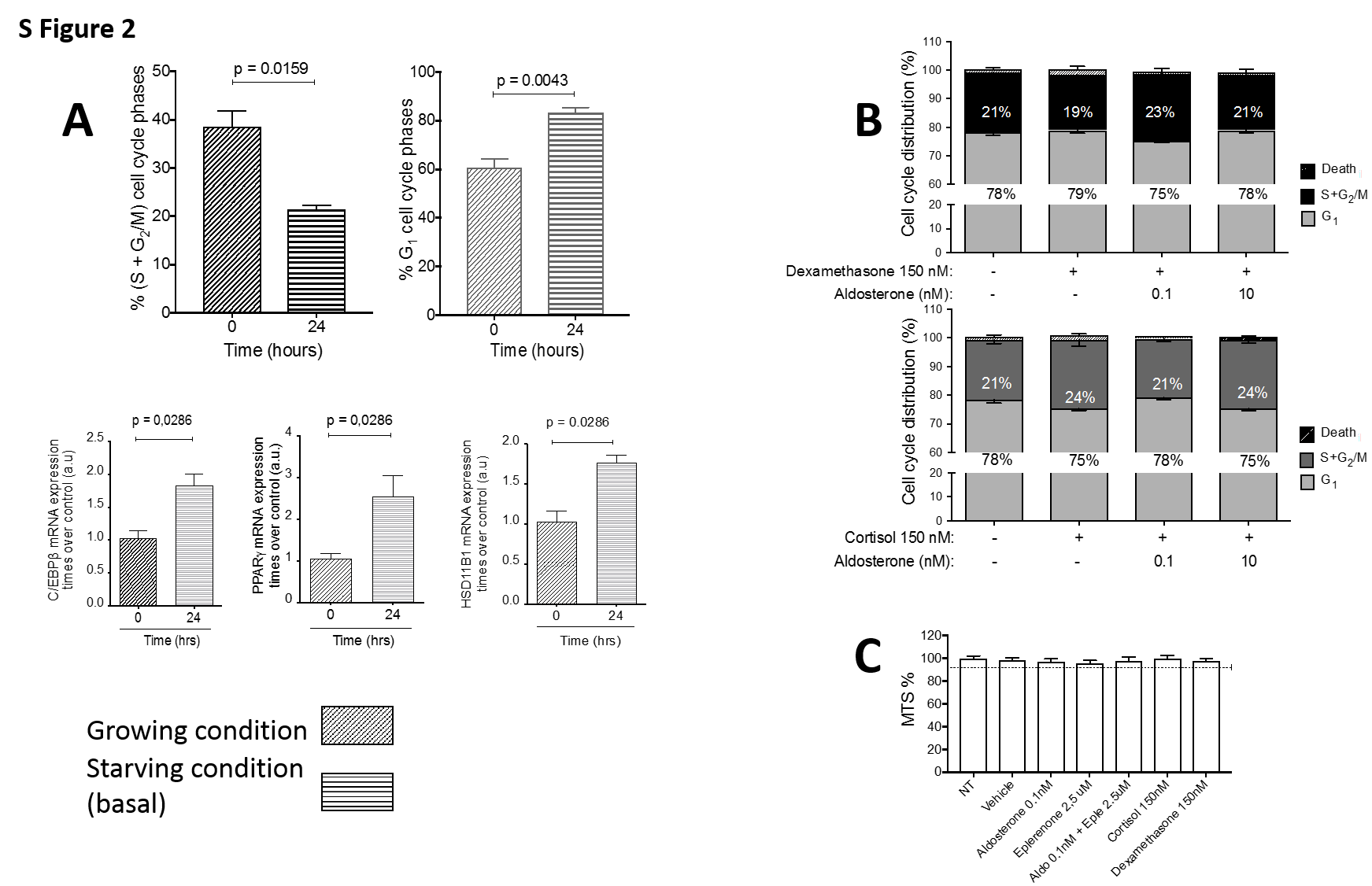

Supplement: Supplementary Figure 2 — F-2S shows the (A) cells that are in (S+G2M) phase and cells in G1, phase before and after arrest by starving with 1% FBS without steroids (FBST). The 1% FBST treatment for 24 h changes the distribution of the cell cycle phases by decreasing cell replication from 40 to 20% (S+G2M) and increasing the arrest (G1) of the total from 60 to 80% of the cell population. Lower panel represents changes in mRNA expression of early and middle commitment genes, before and after arrest by starving with 1% FBS without steroids The mRNA levels of the early C/EBPbeta, PPARgamma or medium 11β-HSDH1 adipogenic genes increased according to the phases of the cell cycle (same cell cycle bar color). Data are presented as the mean ± SEM. P < 0.05 was considered significant. (B) Neither dexamethasone 150 nM (black) nor cortisol 150 nM (gray) reduced the percentage of cells in the proliferative phase, but on the contrary, they increased this percentage. (C) No concentration of drug used affected cell viability. Data are presented as the mean ± SD. Values under 2 standard deviations from the mean were considered toxic (n = 5 per bar). [file Image_2.TIF]

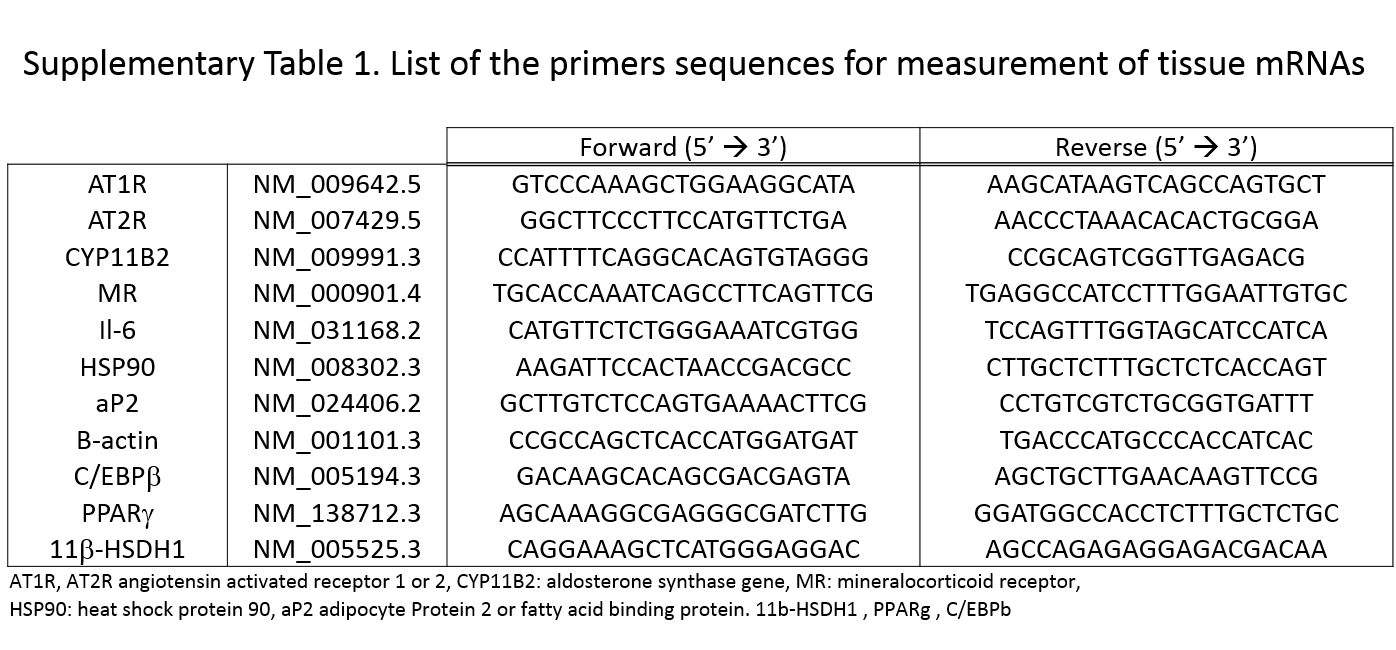

Supplement: Supplementary Table 1 — List of primer sequences for measurement of tissue mRNAs. [file Image_3.TIF]
